# Individual- and provider-level factors associated with colorectal cancer screening in accordance with guideline recommendation: a community-level perspective across varying levels of risk

**DOI:** 10.1186/1471-2458-13-248

**Published:** 2013-03-20

**Authors:** Ryan J Courtney, Christine L Paul, Robert W Sanson-Fisher, Finlay A Macrae, Mariko L Carey, John Attia, Mark McEvoy

**Affiliations:** 1 The Priority Research Centre for Health Behaviour,School of Medicine and Public Health, Faculty of Health, The University of Newcastle, Callaghan, Australia; 2 Department of Colorectal Medicine and Genetics, The Royal Melbourne Hospital, Melbourne, Australia; 3 The Centre for Clinical Epidemiology and Biostatistics, Faculty of Health, The University of Newcastle, Newcastle, Australia; 4 Hunter Medical Research Institute, New Lambton, NSW, 2305, Australia; 5 University of New South Wales, National Drug & Alcohol Research Centre, Sydney, NSW, 2052, Australia

## Abstract

**Background:**

Participation rates in colorectal cancer screening (CRC) are low. Relatively little is known about screening uptake across varying levels of risk and across population groups. The purpose of the current study was to identify factors associated with (i) ever receiving colorectal cancer (CRC) testing; (ii) risk-appropriate CRC screening in accordance with guidelines; and (iii) recent colonoscopy screening.

**Methods:**

1592 at-risk persons (aged 56–88 years) were randomly selected from the Hunter Community Study (HCS), Australia. Participants self-reported family history of CRC was used to quantify risk in accordance with national screening guidelines.

**Results:**

1117 participants returned a questionnaire; 760 respondents were eligible for screening and analysis. Ever receiving CRC testing was significantly more likely for persons: aged 65–74 years; who had discussed with a doctor their family history of CRC or had ever received screening advice. For respondents “at or slightly above average risk”, guideline-appropriate screening was significantly more likely for persons: aged 65–74 years; with higher household income; and who had ever received screening advice. For respondents at “moderately or potentially high risk”, guideline-appropriate screening was significantly more likely for persons: with private health insurance and who had discussed their family history of CRC with a doctor. Colonoscopy screening was significantly more likely for persons: who had ever smoked; discussed their family history of CRC with a doctor; or had ever received screening advice.

**Conclusions:**

The level of risk-appropriate screening varied across populations groups. Interventions that target population groups less likely to engage in CRC screening are pivotal for decreasing screening inequalities.

## Background

Worldwide, colorectal cancer (CRC) is a significant health burden with over one million persons diagnosed annually [1]. The five-year survival rate for localised disease is high, yet few CRCs (less than 40%) are detected at this stage [2]. Many CRC deaths are preventable as screening can reduce incidence through the identification and removal of precancerous polyps [3,4] and increase early detection of disease [5,6]. Australian National Health and Medical Research Council (NHMRC) screening guidelines [7] recommend that asymptomatic persons “at or slightly above average risk” receive either FOBT screening biennially or sigmoidoscopy (preferably flexible) every five years commencing at age 50 years [7]. For persons at “moderately increased risk”, colonoscopy is endorsed every five years starting at age 50 or at an age ten years younger than the age of first diagnosis of bowel cancer in the family, whichever comes first [7]. Endoscopy screening for persons at “potentially high risk” is recommended at least on a five-yearly basis in the NHMRC screening guidelines. However, age at screening commencement, test type and repeat testing interval are dependent on the type of family-specific mutation identified [7].

### Australia’s National Bowel Cancer Screening Program

In Australia, Medicare administers the National Bowel Cancer Screening Program (NBCSP) Register [8]. Medicare selects eligible participants from either the Medicare enrolment records or the Department of Veterans’ Affairs [8]. The NBCSP Register is responsible for mailing of screening invitations and FOBT kits, the recording of participants’ details and the issuing of reminder letters [8]. As part of the NBCSP, participants are encouraged to nominate their usual primary care provider on their participants details form; however, this is not compulsory [8]. The recently re-funded NBCSP offers persons turning 50, 55 and 65 years of age via mail a one-off immunochemical Faecal Occult Blood Test (FOBT) screening invitation [9]. It is important to note that the limited format of the NBCSP (restriction of screening to persons in selected age brackets across the at-risk population), [9] is not consistent with the NHMRC recommendation of biennial FOBT screening of all Australians in the at-risk population [7]. A recent study examining the costs and outcomes from full (rather than limited) implementation of a biennial FOBT screening for adults in the at-risk population (50–74 years) identified a likely mortality reduction of 25% and saving of 500 deaths per year [10].

### Screening participation in Australia

Participation rates in Australia’s NBCSP appear to have reached a plateau since the pilot program’s introduction [9,11-13]. The pilot program received a response rate of 45%, with the roll-out of the NBCSP (with screening offered to persons 50 and 65 years of age) in 2006 receiving a slightly lower rate of 41% [12]. The rate of participation only marginally increased in 2008 to 42.9% when the program widened to offer screening to persons aged 55 years of age [11]. Most recent estimates suggest a similar rate of low participation (40.1%) [9]. The experience in other countries with national screening programs offering repeated FOBT screening to a wider section of the at-risk population suggests that much higher screening rates are achievable. For example, FOBT screening rates in the United Kingdom and Finnish screening programs are currently 52% and 71% respectively [14,15]. In Australia, previous community- based evaluations have also indicated low rates of CRC screening, with 5 to 20% of individuals ever undertaking FOBT [16-18]. The most recent assessment in New South Wales (NSW) indicated that 18% of persons aged over 50 years had undertaken FOBT in the previous five years [19]. In relation to colonoscopy screening in Australia, two population-based assessments in NSW have suggested under screening among persons at elevated levels of risk [16,19].

Given low rates of CRC screening, it is important to identify factors which may influence screening uptake. Previous studies have indicated that the following factors influence CRC screening behaviour: *socio*-*demographic characteristics* (e.g. older age, higher education, higher income); *lifestyle factors* (e.g. smoking history, chronic disease); *family history* (e.g. personal or family history of CRC); *awareness* (e.g. knowledge of CRC and perceived risk of developing CRC) and *health care utilisation* (e.g. usual source of care, number of GP visits, and health care coverage) [20-26]. In the Australian context, a small number of studies have explored determinants of CRC screening uptake [17-19,27,28] with little known about predictors of screening behaviour for persons at varying levels of risk [16]. Since the introduction of the NBCSP in 2006, no community- or population-based assessments in Australia have been conducted. Recent evidence pertaining to FOBT screening uptake and inequalities in participation have been confined to annual NBCSP monitoring reports, which report screening rates among a limited section of the at-risk population (persons 50, 55 and 65 years of age) [9,11-13]. Further, since the NHMRC guidelines’ implementation in 1999, only one study has assessed the predictors of risk-appropriate screening for persons at each level of risk in accordance with guideline-recommendation [16]. The identification of factors associated with risk-appropriate screening is of critical importance for future planning and implementation of tailored CRC screening programs.

This study aimed to assess among a large community-based cohort of at-risk persons (aged 56–88 years), the factors associated with: (1) ever receiving any CRC testing; (2) receiving screening in accordance with screening guidelines; and (3) recent use of colonoscopy screening.

## Methods

The University of Newcastle and Hunter New England Population Health Human Research Ethics Committees granted ethical approval.

### Study population

The Hunter Community Study (HCS) is a longitudinal community cohort aged 55–85 years at baseline in the Hunter Region, New South Wales (NSW), Australia. The sampled population, Hunter Community Study participants are at-risk of developing CRC with clinical practice guidelines recommending all persons commence CRC screening from the age of 50 years [7]. HCS participants were randomly selected from the NSW State electoral roll between December 2004 and December 2007. A modified Dillman recruiting strategy [29] was used for this study, whereby two letters of introduction and an invitation to participate were posted to selected persons. Persons not speaking English and living in a residential aged-care facility were deemed ineligible. Following consent to participate, individuals were asked to complete two self-reported questionnaires and were asked to return these when they attended the HCS data collection centre, at which time a series of clinical measures were obtained. For further details on the HCS cohort and an exhaustive list of measures obtained from HCS participants, see the cohort profile [30]. The HCS cohort profile reflects state and national profiles for gender and marital status but is slightly younger in age [30]. For the current study, a randomly selected sub-sample of the HCS (n = 1592) aged 56–88 years (reflective of age since HCS data collection point) were mailed a questionnaire during November, 2009. The sample size of 1592 was chosen to investigate delay in seeking medical advice for primary symptoms of CRC in additional studies using the HCS cohort [31,32] in order to achieve a power of 80%, so that the proportion of individuals who delay seeking medical advice for symptoms or treatment for CRC will be estimated with 95% confidence intervals.

### Questionnaire

Questions and responses options are presented in the study questionnaire (see Additional file 1). Respondents were asked whether they had ever undertaken FOBT, colonoscopy or sigmoidoscopy. Those ever undertaking each test were asked to specify timing of most recent testing and “Did you have your last X test because you had a symptom” (Yes/ No). All respondents were asked about their family history of CRC (see Additional file 1) to allocate each respondent to a level of risk (see Table 1) in accordance with screening guidelines [7].

**Table 1:** Respondents risk allocation in accordance with CRC screening guidelines

Risk category*	
At or slightly above average risk	• No personal history of bowel cancer.
	• Either no close relatives with bowel cancer or one first-degree or second-degree relative with bowel cancer diagnosed at age 55 years or older.
Moderately increased risk	• One first-degree relative diagnosed before the age of 55 years (without potentially high-risk features listed below), or
	• Two first-degree relatives or one first- and one second-degree relative(s) on the same side of the family (without potentially high-risk features listed below).
Potentially high risk	• Three or more first-degree or a combination of first-degree and second-degree relatives on the same side of the family diagnosed with bowel cancer (suspected HNPCC**), or
	• Two or more first-degree or second-degree relatives on the same side of the family diagnosed with bowel cancer, including the following risk feature: bowel cancer before the age of 50 years or at least one-relative with cancer of the endometrium, ovary, stomach, small bowel, renal pelvis, ureter, biliary tract or brain.

* Risk features potentially placing persons at possible increased risk including: personal history of adenoma, inflammatory bowel disease or suspected familial adenomatous polyposis (FAP) were not assessed in the survey. ** HNPCC (Hereditary non-polyposis colorectal cancer) also known as Lynch syndrome.

### Predictors of CRC screening behaviour

Based on existing literature, the following items selected from the HCS databank, assessed at HCS baseline were investigated: *Socio*-*demographic characteristics*: age, gender, education, marital status, country of birth, household income, retirement, private health insurance status, tobacco or alcohol use; *Clinical characteristics:* number of general practice visits over the past 12 months, previous cancer diagnosis (excluding CRC), body mass index, and presence of a chronic health-condition; and *Psychosocial characteristics:* physical health (SF-36) and mental health (K-10 Kessler Scale). Predictors ascertained at survey completion included: risk category, discussion of family history of CRC with doctor and notification of any “increased risk” (Never discussed/ discussed and informed of possible “increased risk”/ discussed and not informed of any possible “increased risk”), and ever received screening advice from doctor (Yes/No).

### Statistical analysis

Descriptive statistics including frequencies, percentages, means, and standard deviations were used to describe the characteristics of participants. Chi-squared testing was used to assess any responder bias in relation to age, gender, and level of education. “Ever received CRC testing” was calculated by combining the proportion of respondents indicating “Yes” to undertaking any of the following CRC tests (i.e. FOBT, sigmoidoscopy or colonoscopy) divided by the total number of respondents. Respondents who had not provided any information on any of these CRC tests were excluded from analysis. The proportion of respondents screened in accordance with screening guidelines (Yes/ No) was assessed by risk category [7] as follows: “at or slightly above average risk” (FOBT within two years or sigmoidoscopy preferably flexible within five years) and “moderately increased risk/ potentially high risk” (colonoscopy within 5 years). Self reported family history of CRC was used to allocate respondents to a risk category in accordance with screening guidelines. Respondents with missing values relating to type or number of relative(s) diagnosed or age at their diagnosis were excluded from analysis. Multiple logistic regression modelling was used to assess the association between socio-demographic, clinical and psychosocial characteristics and each study outcome: ever received CRC test, screened in accordance with guidelines for level of risk (“at or slightly above average risk” and “moderately/ potentially increased risk”), and recent colonoscopy screening (within previous five years). Variables with a *p* value <.25 following simple logistic regression analysis (see Additional file 2) were entered into a stepwise multiple logistic regression analysis. Forward and Backward stepwise regression was used on complete cases so that the likelihood ratio test could be used to determine the best fit from one iteration to the next. Both the forward selection and backward elimination regression analysis produced equivalent results. Results from the backward stepwise elimination model were reported. Variables that met the significance cut point (*p* value < .05) were included in the final model. Data were analysed using STATA 11 (STATA, Texas, USA).

## Results

### Characteristics of the sample

Of the 1592 mailed surveys, 1117 respondents completed and returned a survey (response rate = 70%). Respondents previously diagnosed with CRC (n = 24) or reporting they had undergone major abdominal surgery (n = 8) were excluded from analysis, leaving a total sample of 1085 participants with data. Responder bias was assessed on the following key characteristics: gender, age (56–64, 65–74, 75–88), and education (secondary schooling completed, secondary schooling not competed, trade qualification or TAFE, university or other tertiary education and other). Pearson’s χ2 test found no significant differences relating to gender (χ2 = .09; df =1 ; *p* = .76 ) or education (χ2 = 8.75; df = 4; *p* = .07). However, for age, respondents were significantly more likely to be of a younger age compared to non-respondents (χ2 = 11.12; df = 2; *p* < .01).

### Inclusion and exclusion for CRC screening

Respondents were classified as asymptomatic and eligible for screening if they had *not reported* the following: undertaking FOBT, sigmoidoscopy or colonoscopy due to a symptom episode in the previous five years; any bowel related condition; previous history of CRC; or consulting a doctor for their first primary symptom episode of CRC (rectal bleeding or change in bowel habit) in the previous five years. Following exclusion of such persons, a total sample of 777 asymptomatic persons were identified. 17 asymptomatic persons were excluded from analysis due to insufficient family history information available to derive a level of risk in accordance with CRC screening guidelines. Table 2 describes the characteristics of respondents allocated to a level of risk in accordance with screening guidelines (n = 760) and eligible for analysis.

**Table 2:** Socio-demographic, clinical and psychosocial characteristics of the asymptomatic study population (n = 760)

**Characteristic**		**n**	%**
Socio-demographic			
Gender*	Male	363	48
	Female	397	52
Age (years)*	56-64	317	42
	65-74	268	36
	75-88	168	22
Marital status*	In Relationship	575	79
	Not in Relationship	155	21
Education*	Secondary schooling (not-completed)	164	22
	Secondary schooling (completed)	162	22
	Trade qualification or TAFE:	189	26
	University or other tertiary study	181	25
	Other or not applicable	33	5
Household income before tax ($)*	<= 39, 999	389	57
	40, 000 – 69, 999	152	22
	>= 70,000	146	21
Country of birth*	Australian	619	89
	Other	76	11
Retired*	Yes	453	62
Private health insurance*	No-coverage	191	26
	Coverage	531	74
Alcohol*	Drink days per month	Mean =11	SD =11
Smoking*	Ever	346	48
	Never	382	52
Clinical			
Number of GP visits over the past 12 months*	None to twice	187	25
	Three to six	411	56
	> six	137	19
Previous cancer (excluding CRC)*	Yes	163	22
Risk Category	At or slightly above average risk	707	93
	Moderately increased risk	34	4
	Potentially high risk	19	3
Discussion of family history of CRC with doctor	Never discussed	523	70
	Discussed, informed of ‘increased risk’	133	18
	Discussed, not informed of ‘increased risk’	90	12
Ever received screening advice from doctor	Yes	195	28
BMI*	< 18.5	7	1
	18.5 – 25	130	19
	> 25	555	80
Chronic health condition*	Yes	554	76
Psychosocial			
SF-36 (physical health score)*		Mean = 52	SD = 9
K-10 (mental health score)*	Low or no risk (10–15)	555	75
	Medium to high risk (16 +)	184	25

* Participant data collected at HCS baseline. ** Percentage of responses (excluding any missing values).

### Ever received CRC testing

Overall, 63% (475/ 760) of respondents allocated to a level of risk had ever received any CRC testing (FOBT/ sigmoidoscopy or colonoscopy). The proportion ever undertaking CRC testing by risk category were: “at or slightly above average risk” 61% (431/ 707), “moderately increased risk” 82% (28/34) and “potentially high risk” 84% (16/19). Simple logistic regression analyses identified items with a *p* value < .25 which were entered into multiple logistic regression modelling. Table 3 presents the results of this analysis. Persons significantly more likely to have ever been tested for CRC were aged between 65–74 years of age and had ever received screening advice from a doctor. Persons who had discussed their family history of CRC with a doctor, irrespective of whether they were informed of any possible “increased risk”, were significantly more likely to be tested than those never having discussed their family history with a doctor.

**Table 3:** Multiple logistic regression model (n = 689)* of factors associated with ever receiving CRC testing

**Characteristic**	n** (%)	**OR (95% CI)**	***p *value**
Age (years)			
56-64	172 (57)	1	
65-74	171 (71)	2.00 (1.36, 2.97)	<.001
75-88	86 (59)	1.27 (.82, 1.96)	.286
Discussion of family history of CRC with doctor			
Never discussed	248 (52)	1	
Discussed/ informed of possible ‘increased risk’	114 (90)	4.13 (2.12, 8.05)	<.001
Discussed/ not informed of possible increased ‘risk’	67 (83)	3.78 (2.02, 7.05)	<.001
Ever received screening advice from doctor			
No	264 (53)	1	
Yes	165 (88)	3.59 (2.11, 6.08)	<.001

* Respondents excluded from model due to missing values (n = 71).

** Number of respondents ever received CRC testing.

### CRC screening in accordance with national screening guidelines

Of the 707 respondents “at or slightly above average risk”, 697 provided information on either FOBT or sigmoidoscopy screening, with 21% (145/697) of respondents screened in accordance with national screening recommendation (see Table 4). Screening in accordance with guideline recommendation was significantly more likely to occur for “at or slightly above average risk” persons: aged 65–74 years of age; with a higher household income (> $70, 000 compared to <= $39, 999); and who had ever received screening advice from a doctor.

**Table 4:** Multiple logistic regression model (n = 586*) of factors associated with screening in accordance with guidelines for persons “at or slightly above average risk”

**Characteristic**	n** (%)	**OR (95% CI)**	***p *value**
Age (years)			
56-64	46 (17)	1	
65-74	59 (29)	2.71 (1.64, 4.49)	<.001
75-88	21 (18)	1.57 (.83, 2.96)	.166
Annual household income before tax ($)			
<= 39, 999	60 (18)	1	
40, 000 – 69, 999	27 (21)	1.28 (.74, 2.21)	.380
>= 70,000	39 (30)	2.59 (1.50, 4.49)	<.001
Ever received screening advice from doctor			
No	72 (16)	1	
Yes	54 (38)	3.00 (1.95, 4.64)	<.001

* Respondents excluded from model due to missing values (n = 111).

** Number of respondents screened in accordance with guidelines.

Of the 53 respondents at “moderately increased risk/ potentially high risk” and providing colonoscopy screening information, 45% (24/53) were screened in accordance with screening recommendation. Screening in accordance with guideline recommendation (colonoscopy within 5 years) was significantly more likely to occur for persons who had private health insurance and who had discussed their family history of CRC with a doctor (see Table 5).

**Table 5:** Multiple logistic regression model (n = 50*) of factors associated with screening in accordance with guidelines for persons at “moderately increased risk/ potentially high risk”

**Characteristic**	n** (%)	**OR (95% CI)**	***p *value**
Private health insurance			
No-coverage	3 (18)	1	
Coverage	19 (58)	8.46 (1.58, 45.39)	.013
Discussion of family history of CRC with doctor			
Never discussed	3 (14)	1	
Discussed/ informed of ‘increased risk’	17 (68)	16.34 (3.19, 83.55)	.001
Discussed/ not informed of increased ‘risk’	2 (67)	17.50 (.83, 368.04)	.066

* Respondents excluded from model due to missing values (n = 3).

** Number of respondents screened in accordance with guidelines.

### Recent colonoscopy screening irrespective of level of risk

Of the 760 respondents allocated to a level of risk, 752 respondents provided colonoscopy screening information, with 16% (124/752) of respondents receiving a colonoscopy within the previous five years. Table 6 presents the multiple logistic regression model for recent colonoscopy screening (within 5 years) irrespective of level of risk. Recent colonoscopy screening was significantly more likely for persons: that had ever smoked; discussed their family history of CRC with a doctor regardless of whether informed of “increased risk”; and who had ever received screening advice from a doctor.

**Table 6:** Multiple logistic regression model (n = 659)* for colonoscopy screening (within 5 years) irrespective of level of risk

**Characteristic**	n** (%)	**OR (95% CI)**	***p *value**
Smoking			
Never	47 (14)	1	
Ever	63 (20)	1.89 (1.15, 3.10)	.012
Discussion of family history of CRC with doctor			
Never discussed	30 (6)	1	
Discussed/ informed of ‘increased risk’	65 (53)	7.20 (3.95, 13.09)	<.001
Discussed/ not informed of increased ‘risk’	15 (20)	2.30 (1.11, 4.76)	.002
Ever received screening advice from doctor			
No	31 (6)	1	
Yes	79 (44)	4.60 (2.65, 8.00)	<.001
Private health insurance			
No-coverage	17 (10)	1	
Coverage	93 (19)	1.84 (.97, 3.50)	.062

* Respondents excluded from model due to missing values (n = 93).

** Number of respondents receiving recent colonoscopy screening.

## Discussion

The behaviour of health care providers including discussing family history of CRC and making a CRC screening recommendation were predictors of CRC screening uptake, screening in accordance with guideline recommendation and recent colonoscopy screening. Individual and lifestyle characteristics associated with some but not all of the above CRC screening outcomes included: higher household income, age, private health insurance and smoking.

### Factors associated with CRC screening behaviour

#### Health care provider recommendation for CRC screening

The variable most strongly associated with CRC testing and screening was receiving screening advice from a health care provider. This finding is consistent with previous literature which identified physician encouragement as an important predictor of CRC screening [20,22,24]. Strategies such as general practitioner screening invitations sent to patients [33,34] and provider reminder/ prompt systems are effective in increasing the likelihood of screening participation [35]. Relatively little is known about how well CRC screening is included within practice-based reminder systems [36,37]. Improvement in the systematic delivery of CRC screening advice and implementation of effective primary-care based interventions are likely to be beneficial.

#### Discussion of family history of CRC with doctor

Persons who had discussed their family history of CRC regardless of whether they were informed of possible “increased risk” were more likely to be ever tested, screened in accordance with guideline recommendation (“moderately/ potentially high risk” persons) and screened recently using colonoscopy. Such findings highlight the importance of family history of CRC assessment in the primary care setting. Studies of cancer risk assessment tools suggest that such an approach is feasible and effective for collection of family history of cancer information, automation of familial risk stratification and risk-appropriate screening advice [38,39].

#### Age

Among persons who were “at or slightly above average risk”, those who were older were more likely to ever be tested and screened in accordance with CRC screening guidelines, as per previous studies [19,24,40,41]. The literature suggests that an inverted U-relationship is observed, with the lowest rates of CRC screening participation found among persons 50–55 years and 70–80 years of age [24,42]. Further, it has also been demonstrated that non-adherence to repeat FOBT screening is evident for persons aged less than 65 years [43]. Given the rapid increase in CRC risk after age 50, the targeting of those aged 50–65 years is of particular importance if optimal screening rates are to be achieved.

#### Household income

A gradient in CRC screening participation has been identified worldwide related to socio-economic status [20,24,42]. Patient screening preference is sensitive to out of pocket expenses [44] with lower participation among disadvantaged persons [21,24]. In the current study, persons “at or slightly above average risk” with household incomes above >$70,000 were more likely to be screened in accordance with guideline recommendation, suggesting a need for interventions facilitating CRC screening among low income households. In Australia, annual NBCSP monitoring reports and cross-sectional analysis of participation in this program have also indicated significantly lower levels of CRC screening participation among persons from the most deprived socio-economic quintiles in the population [9,11-13,45]. It is important to consider that this disparity in participation among differing socio-economic groups in Australia and the United Kingdom (UK) occurs in the presence of universal programs offering FOBT screening at no cost [45]. A similar socio-economic gradient in screening participation has also been identified for cervical and breast cancer screening programs [21]. These findings, lend support to the suggestion that direct economic barriers alone do not explain the socio-economic differential in participation [42]. Previous studies have indicated that the provision of universal healthcare or re-imbursement for the cost of screening has little appreciable effect on eliminating equality in screening participation [46-49]. It appears that some health service access hurdles appear to dominate among disadvantaged persons [42,50]. Additionally, it is argued that there are distinct differences in healthcare-seeking behaviour and beliefs among persons from lower socio-economic groups, compared with those from higher socio-economic groups [42,50]. Taken together, this suggests that strategies to improve equality in screening must recognise factors other than financial coverage [42]. It is paramount that future formative research examines the beliefs and barriers to CRC screening participation among lower socio-economic persons, to assist in the development of effective interventions [51,52]. When considering future decisions about Australia’s NBCSP, it is critical that policy makers consider the reasons for non-screening compliance among persons from lower socio-economic status if increased equality in participation is to eventuate.

#### Private health insurance

Persons with private health insurance were marginally more likely to have recent colonoscopy screening. Previous literature indicates that private health insurance is a strong predictor of CRC screening [19,24,53]. Importantly, the current finding that private health insurance is a predictor for all colonoscopies but not for colonoscopies in accordance with guidelines casts some doubt on the process by which colonoscopies are decided. There is a pressing need to identify ways to improve guideline-appropriate use of colonoscopies.

#### Smoking

Previous studies have indicated that smokers tend to be less compliant with CRC screening [19,24]. The increased likelihood of recent colonoscopy screening among persons who had ever smoked in the current study may be due to health providers’ identification of this risk factor and provision of colonoscopy screening. Alternatively, ex-smokers who have already made a health behaviour change in their lives may have an increased motivation for health screening compared to persons who had never smoked.

### Limitations

It should be acknowledged that responder bias place some limitations on the interpretation of findings and their generalisability. For the most part, the HCS is a representative sample of the Hunter Region and NSW in comparison to Census data on a number of demographic characteristics, with the exception of age [30]. Analysis of responders’ bias identified that responders were more likely to be younger than non-responders from the HCS cohort. In addition, persons living in residential care facilities and those from non-English speaking background were excluded in this study. Previous literature has indicated that non-English speaking persons are significantly less likely to undertake CRC screening [11-13,19]. Future population based CRC screening programmes could benefit from strategies to improve CRC screening in non-English speaking persons and migrant groups [19]. For respondents undertaking FOBT, type of test undertaken (guaiac or immunochemical) and the outcome of testing (negative/ positive) was not assessed, and this should be considered when interpreting the rate of colonoscopy screening (previous five years), as it may be an independent predictor for seeking a colonoscopy.

While it must be acknowledged that self-reported screening behaviour may have some recall bias, it has been shown to have reasonable agreement with physician report and relatively high levels of sensitivity and specificity [54,55]. Although two new studies and a recent meta-analysis have indicated high levels of sensitivity and specificity for self-reported screening behaviour, [56-58] it is important to recognise that accuracy of self-reported screening varies across patient characteristics and test modalities [20]. A meta-analysis of validation studies on self-reported CRC cancer screening use in the United States found that self-report *versus* documented history of screening had a high level of specificity (endoscopy .90 and FOBT .78) and sensitivity (endoscopy .82 and FOBT .79) [56].

To enhance recall, the current study adopted descriptions of CRC tests to increase the accurate recall of CRC testing. Self-reported family history of CRC was used to assign level of risk. Previous studies have indicated self-reported family history of CRC has relatively high levels of sensitivity and specificity for reporting of affected first-degree relatives [59-61]. However, the level of accuracy is substantially reduced for affected second-degree relatives [59-61]. The accuracy of reporting of age at diagnosis in CRC- affected relatives has not been identified in the literature. Further, the extent to which misleading family history information reorients risk classification for relatives following verification is largely unknown [61]. Therefore, it is possible that the classification of risk may be inaccurate for a small proportion of study respondents given the limitation of no objective verification of self reported family history. For respondents at “moderately increased/ potentially high risk”, level of risk may have been overestimated for a small minority of respondents. For respondents indicating that both a first-degree relative and second-degree relative were diagnosed with CRC, an assumption was made that both relatives were diagnosed on the same side of the family. This assumption may have over-estimated the level of risk for some respondents.

Finally, the current study is subject to limitations associated with stepwise regression analysis including: parameter estimation, error rate estimation and reliance on a single best model [62-65]. Conducting stepwise regression models with a large number of independent variables and a small sample size, may over-estimate model fit [63,64]. However, this limitation is difficult to overcome when applying statistical modelling techniques to identify predictors associated with rare occurrences in the population i.e. persons at increased familial risk of disease. It is conceivable that future large scale population-based studies examining CRC screening behaviour across varying levels of familial risk will assist in further defining the predictors of risk-appropriate CRC screening.

## Conclusions

Empirical randomised controlled trial evidence suggests that the offering of repeated (annual or biennial) FOBT screening to the at-risk population is effective in reducing mortality and incidence associated with CRC [3,6,66,67]. In Australia, there is a pressing need for expansion of the NBCSP and offering of repeated screening to the at-risk population, if the CRC-related disease burden and economic cost is to be reduced [68]. Multiple strategies and messages targeting specific demographic groups as well as healthcare provider factors are needed if increases in overall screening participation are to be achieved. A pressing issue is the reduction of inequalities in CRC screening participation to reduce inequality in health outcomes. High quality research is required to unravel the barriers to CRC screening, as well as the mechanisms by which screening inequalities are maintained.

## Competing interests

The authors declare that there are no conflicts of interest.

## Authors’ contributions

RJC, CLP, RSF were responsible for study design, data collection, analysis and drafting. All authors contributed to the interpretation of results and writing of this manuscript. All authors read and approved the final manuscript.

## Pre-publication history

The pre-publication history for this paper can be accessed here:

http://www.biomedcentral.com/1471-2458/13/248/prepub

## Supplementary Material

Additional file 1 Early Detection of Bowel Cancer Study. Click here for file

Additional file 2 Simple logistic regression analysis for socio-demographic, lifestyle, clinical and psychosocial characteristics associated with CRC testing/ screening outcomes. Click here for file
