# Supplementary material for: Individual- and provider-level factors associated with colorectal cancer screening in accordance with guideline recommendation: a community-level perspective across varying levels of risk
Source: BMC Public Health. 2013 Mar 20;13:248. doi: 10.1186/1471-2458-13-248 (PMC3607924; doi:10.1186/1471-2458-13-248)
Supplement: Additional file 2 — Simple logistic regression analysis for socio-demographic, lifestyle, clinical and psychosocial characteristics associated with CRC testing/ screening outcomes. [file 1471-2458-13-248-S2.doc]

**Supplementary material B**

Simple logistic regression analysis for socio-demographic, lifestyle, clinical and psychosocial characteristics associated with CRC testing/ screening outcomes.

|  | Ever received CRC test | | Screening in accordance with guideline  (“At or slightly above average risk”) | | Screening in accordance with guideline  (“Moderately/ potentially high risk”) | | Recent colonoscopy screening  (irrespective of risk category) | |
| --- | --- | --- | --- | --- | --- | --- | --- | --- |
|  | OR (95% CI) | *p*-value* | OR (95% CI) | *p*-value | OR (95% CI) | *p*-value | OR (95% CI) | *p*-value |
| Socio-demographic characteristics |  |  |  |  |  |  |  |  |
| Gender |  |  |  |  |  |  |  |  |
| Female | .71 (.53, .95) | **.023** | .57 (.39, .82) | **.003** | .63 (.18, 2.23) | .477 | .94 (.64, 1.39) | .772 |
| Age (years) |  |  |  |  |  |  |  |  |
| 56-64 | 1 |  | 1 |  | 1 |  | 1 |  |
| 65-74 | 1.80 (1.27, 2.54) | **.001** | 1.82 (1.21, 2.75) | **.004** | 1.02 (.30, 3.44) | .976 | 1.23 (.79, 1.90) | .348 |
| 75-88 | 1.10 (.76, 1.62) | .607 | .93 (.55, 1.58) | .799 | .20 (.36, 1.16) | **.073** | .97 (.57, 1.64) | .907 |
| Marital status |  |  |  |  |  |  |  |  |
| In Relationship | 1 |  | 1 |  | 1 |  | 1 |  |
| Not in Relationship | .74 (.51, 1.05) | **.096** | .57 (.34, .95) | **.031** | .26 (.05, 1.39) | **.116** | .73 (.43, 1.22) | **.229** |
| Education |  |  |  |  |  |  |  |  |
| Secondary schooling (not-completed) | 1 |  | 1 |  | 1 |  | 1 |  |
| Secondary schooling (completed) | 1.08 (.69, 1.69) | .717 | 1.71 (.94, 3.10) | **.077** | .44 (.08, 2.46) | .353 | .83 (.45, 1.53) | .547 |
| Trade qualification or TAFE: | 1.17 (.76, 1.80) | .463 | 1.81 (1.02, 3.20) | **.042** | .83 (.15, 4.63) | .835 | .86 (.48, 1.55) | .625 |
| University or other tertiary study | 1.58 (1.01, 2.47) | **.043** | 1.79 (1.00, 3.18) | **.049** | 1.00 (.19, 5.07) | 1.00 | 1.32 (.76, 2.30) | .320 |
| Other or not applicable | 1.06 (.49, 2.28) | .876 | 1.46 (.54, 4.00) | .455 | .50 (.03, 7.45) | .615 | 1.14 (.43, 3.05) | .786 |
| Household income before tax ($) |  |  |  |  |  |  |  |  |
| <= 39, 999 | 1 |  | 1 |  | 1 |  | 1 |  |
| 40, 000 – 69, 999 | 1.33 (.90, 1.98) | **.149** | 1.26 (.78, 2.04) | .349 | 1.07 (.23, 4.84) | .933 | 1.00 (.60, 1.66) | .991 |
| >= 70,000 | 1.30 (.87, 1.93) | **.201** | 1.83 (1.15, 2.89) | **.010** | 1.07 (.23, 4.84) | .933 | 1.01 (.60, 1.69) | .968 |
| Country of birth |  |  |  |  |  |  |  |  |
| Australian | 1 |  | 1 |  | 1 |  | 1 |  |
| Other | .54 (.33, .86) | **.011** | .75 (.39, 1.41) | .374 | 1.23 (.07, 20.76) | .847 | .73 (.36, 1.46) | .369 |
| Retired |  |  |  |  |  |  |  |  |
| Yes | 1.28 (.94, 1.74) | **.012** | 1.15 (.78, 1.70) | .469 | .88 (.28, 2.74) | .829 | 1.08 (.72, 1.62) | .712 |
| No | 1 |  |  |  |  |  | 1 |  |
| Private health insurance |  |  |  |  |  |  |  |  |
| No-coverage | 1 |  | 1 |  | 1 |  | 1 |  |
| Coverage | 1.51 (1.08, 2.13) | **.015** | 1.33 (.85, 2.08) | **.207** | 5.91 (1.43, 24.43) | **.014** | 2.20 (1.29, 3.74) | **.004** |
| Alcohol |  |  |  |  |  |  |  |  |
| Drink days per month | 1.02 (1.00, 1.03) | **.019** | 1.02 (1.00, 1.04) | **.008** | 1.00 (.96, 1.05) | .824 | .99 (.98, 1.01) | .581 |
| Smoke |  |  |  |  |  |  |  |  |
| Never | 1 |  | 1 |  | 1 |  | 1 |  |
| Ever | 1.11 (.82, 1.50) | .501 | 1.11 (.76, 1.61) | .592 | 3.15 (.98, 10.14) | **.054** | 1.47 (.98, 2.17) | **.058** |
| Clinical characteristics |  |  |  |  |  |  |  |  |
| Number of GP visits over the past 12 months |  |  |  |  |  |  |  |  |
| None to twice | 1 |  | 1 |  | 1 |  | 1 |  |
| Three to six | 1.30 (.91, 1.85) | **.148** | .84 (.54, 1.29) | .415 | 4.92 (.89, 27.32) | **.068** | 2.00 (1.18, 3.39) | **.009** |
| > six | 1.29 (.82, 2.02) | .279 | .77 (.44, 1.37) | .384 | 3.42 (.52, 22.80) | **.202** | 1.62 (.84, 3.10) | **.149** |
| Previous Cancer (excluding CRC) |  |  |  |  |  |  |  |  |
| Yes | 1.34 (.93, 1.94) | **.123** | .93 (.59, 1.45) | .740 | 1.39 (.30, 6.30) | .670 | 1.59 (1.02, 2.47) | **.039** |
| No | 1 |  | 1 |  | 1 |  | 1 |  |
| Risk Category |  |  |  |  |  |  |  |  |
| At or slightly above average risk | 1 |  |  |  |  |  | 1 |  |
| Moderately increased risk | 2.99 (1.22, 7.31) | **.016** | - |  | - |  | 4.19 (2.05, 8.57) | **.000** |
| Potentially high risk | 3.41 (.99, 11.83) | **.053** |  |  |  |  | 6.66 (2.64, 16.79) | **.000** |
| Discussion of family history of CRC with doctor |  |  |  |  |  |  |  |  |
| Never discussed | 1 |  | 1 |  |  |  | 1 |  |
| Discussed, informed of ‘increased risk’ | 7.90 (4.42, 14.11) | **.000** | 1.54 (.93, 2.54) | **.091** | 10.69 (2.74, 41.74) | **.001** | 13.64 (8.44, 22.01) | **.000** |
| Discussed, not informed of ‘increased risk’ | 3.99 (2.29, 6.96) | **.000** | 2.34 (1.42, 3.86) | **.001** | 9.5 (.68, 132.00) | **.094** | 3.35 (1.81, 6.21) | **.000** |
| Ever received screening advice from doctor |  |  |  |  |  |  |  |  |
| No | 1 |  |  |  | 1 |  | 1 |  |
| Yes | 6.59 (4.12,10.54) | **.000** | 3.65 (2.44, 5.46) | **.000** | 16.5 (1.91, 142.49) | **.011** | 9.18 (5.89, 14.30) | **.000** |
| BMI |  |  |  |  |  |  |  |  |
| < 18.5 | .45 (.09, 2.11) | .314 | .82 (.09, 7.34) | .857 | - |  | - |  |
| 18.5 - 25 | 1 |  | 1 |  |  |  | 1 |  |
| > 25 | 1.12 (.76, 1.67) | .557 | 1.14 (.69, 1.89) | .592 | .95 (.24, 3.81) | .942 | 1.14 (.68, 1.91) | .608 |
| Comorbidity |  |  |  |  |  |  |  |  |
| No | 1 |  | 1 |  | 1 |  | 1 |  |
| Yes | 1.08 (.76, 1.53) | .682 | 1.20 (.77, 1.89) | .419 | .51 (.12, 2.20) | .368 | 1.13 (.70, 1.82) | .613 |
| Psychosocial characteristics |  |  |  |  |  |  |  |  |
| SF-36 (physical health score) | 1.01 (.99, 1.03) | .202 | 1.02 (.99, 1.04) | **.061** | 1.01 (.96, 1.07) | .654 | 1.00 (.99, 1.03) | .475 |
| K-10 (mental health score) |  |  |  |  |  |  |  |  |
| Low or no risk (10-15)_ | 1 |  | 1 |  | 1 |  | 1 |  |
| Medium to high risk (16 +) | .83 (.59, 1.17) | .290 | .49 (.30, .81) | **.005** | .79 (.24, 2.56) | .691 | 1.187(.75, 1.82) | .484 |

** p* values < .25 included in multiple regression model bold
